# *Bifidobacterium lactis* in Treatment of Children with Acute Diarrhea. A Randomized Double Blind Controlled Trial

**DOI:** 10.3889/oamjms.2015.088

**Published:** 2015-08-07

**Authors:** Neveen Helmy Abou El-Soud, Reem Nabil Said, Dalia Sayed Mosallam, Nahla Abdel Moniem Barakat, Mohamed Ahmed Sabry

**Affiliations:** 1 *Complementary Medicine Department, Medical Researches Division, National Research Center, El-Behouth Street, 12311 Cairo, Egypt*; 2 *Pediatric Department, Faculty of Medicine, Cairo University, Cairo, Egypt*; 3 *Pediatric Department, National Research Center, El-Behouth Street, 12311 Cairo, Egypt*

**Keywords:** probiotics, *Bifidobacterium lactis*, acute diarrhea, children

## Abstract

**BACKGROUND:**

Probiotics are becoming increasingly popular treatment for children diarrhea. Although there are several probiotic strains potentially useful, researches were often limited to certain strains.

**AIM:**

To test *Bifidobacterium lactis* on morbidity of acute diarrhea in children less than 2 years.

**SUBJECTS AND METHODS:**

A randomized double-blind controlled clinical trial was conducted in 50 children (1 - 23 months) admitted with acute diarrhea to the Pediatric Hospital, Cairo University and were randomly assigned to receive in addition to usual treatment of diarrhea according to WHO guidelines; one of two treatments either milk formula non-supplemented (n = 25) or supplemented (n = 25) with *Bifidobacterium lactis* 14.5 × 10^6^ CFU/100 ml daily for one week. Primary outcomes were frequency and duration of diarrhea and hospital stay. Secondary outcomes were duration of fever and vomiting episodes. Safety and tolerance were also recorded.

**RESULTS:**

On admission, patients’ characteristics of both groups (50 cases) were similar. For children who received the probiotics for one week; mean duration of diarrhoea was shorter than in controls (3.12 ± 0.92 vs. 4.10 ± 0.94 days) (P = 0.02), number of motions per day was less than in controls (3.96 ± 0.62 vs. 4.46 ± 0.85) (P = 0.04) and discharge from hospital <2 days was more frequent than in controls (72% vs. 44%) (P = 0.048). There was no effect on fever (P = 0.63) or vomiting (P = 0.54).

**CONCLUSION:**

*Bifidobacterium lactis* probiotics in supplemented milk formula decreased significantly frequency, duration of diarrhea, and hospital stay than usual treatment alone in children with acute diarrhea. Additional researches on other uncommon local probiotic species should be encouraged.

## Introduction

The use of probiotics, discussed primarily in the context of alternative medicine and now entering mainstream medicine, only a few have been confirmed in well-designed controlled trials [1].

Probiotics according to FAO/WHO [2] are live microorganisms which when administered in adequate amounts confer a health benefit on the host. Research studies on probiotics vary according to type of genera, species and strain used beside additional intervention characteristics such as the dose, potency, treatment duration, viability of the organism, as well as the combination of strains [3]. Of the probiotics derived from food sources (cultured milk products); lactic acid bacteria (e.g., *Lactobacillus* and *Bifidobacterium*), *Saccharomyces boulardii* (a non-pathogenic strain of yeast), *Enterococcus* *faecium*, *Streptococcus thermophiles, Pediococcus acidilactici, Bacillus coagulans* and a non-pathogenic strain of *Escherichia coli* (e.g., *E. coli* Nissle, 1917). In children, probiotics were recommended to be used in allergies, inflammatory bowel disease, irritable bowel syndrome and necrotizing enterocolitis [4]. They are commonly used in treatment and prevention of acute diarrhea [5, 6]. Application of probiotics in treating acute children diarrhoea has increasingly become a subject of research interest. It has been reported that probiotics decrease the duration of diarrhoea and fever significantly in children [7].

The strongest evidence of a beneficial effect of probiotics has been established with *L. rhamnosus* *GG* [8] and *B. lactis* *BB-12* [9] for prevention and *Lactobacillus reuteri SD2222* for treatment of acute rotavirus diarrhea in children [10]. Common strains used to treat diarrhea include: *L. rhamnosus* [11], *L. acidophilus* [12], *L. casei* [13], *L. reuteri* [14], *B. bifidum* [15], *B. longum* [16], *S. boulardi* [17], *E. faecium* [18]. Probiotics for diarrhea exert their action possibly through direct or indirect mechanisms including immune modulation, inhibition of pathogenic bacteria, enhancing barrier function and production of antimicrobial agents [19].

*Bifidobacteria* are particularly attractive as probiotics agent because they constitute the predominant colonic flora of breastfed infants and are thought to play a role in the decreased incidence of diarrhea in breastfed infants [20].

However, there are few studies investigating the role of this probiotics. In this study we investigated the effect of using 14.5 × 10^6^ CFU/100 ml daily of *Bifidobacterium lactis* in supplemented milk formula for children acute diarrhea.

## Subjects and Methods

The study protocol was approved by the ethical committee of the National Research Center and Pediatric Department of Cairo University. Informed written consent was obtained from parents of children enrolled in the study.

Fifty children 1 to 23 months of age with acute diarrhea due to gastroenteritis were enrolled in this prospective, double blind, randomized study conducted on admitted children with acute diarrhea to the Paediatric Hospital, Cairo University between July and September 2014. Diarrhoea was defined as passage of three or more loose stools in the last 24 hours [21].

Children with severe malnutrition (weight for height < 3 SD of WHO charts), dysentery (presence of visible blood in stools), clinical evidence of co-existing acute systemic illnesses (e.g. meningitis, sepsis, pneumonia) and clinical evidence of chronic disease (e.g. chronic gastrointestinal disease, chronic liver disease, and chronic renal disease) were excluded from the study. Subjects in whom probiotics were used in the preceding four weeks or if antibiotics were used for current episode of diarrhoea, were also excluded from the study.

Each patient’s demographic data, medical history, feeding pattern and stooling characteristics were recorded. Feeding parameters included number of meals per day, response to food (on a 1–5 scale), daily formula volume, and daily number of regurgitation, degree of dehydration, and the symptoms associated with gastroenteritis (duration of diarrhea, number of motions, fever, vomiting), duration of hospital stay, growth parameters, safety and tolerance were also recorded. Clinical assessment of dehydration was done according to clinical dehydration scale [22].

Patients with severe dehydration had been excluded from the study. The patients estimated as moderate dehydration with no oral tolerance had needed to be hospitalized. From each patient with diarrhea a stool sample was analyzed for routine bacterial cultures (including *Salmonella*, *Shigella*, *Campylobacter*, and *Yersinia* species, excluding toxigenic *Escherichia coli*), rotavirus, ova and parasites, including *Cryptosporidium*.

### Randomization

All included children were randomized to receive either milk formula with *Bifidobacterium Lactis* (*B. lactis* group) or no probiotics milk formula (control group) using serially numbered sealed opaque envelopes.

### Intervention

All the studied patients with acute diarrhea received the usual treatment according to WHO guidelines [20]; in addition the *B. lactis* group received formula with *Bifidobacterium Lactis* using 14.5 × 10^6^ CFU (colony forming unit)/100 ml daily for 7 days and the control group received milk formula with no *Bifidobacterium Lactis* for 7 days. During the study period, patients were followed up in the hospital; to elicit frequency of diarrhea, episodes of vomiting or fever per day and the total duration of diarrhea.

### Statistical analysis

Data was grouped as patients and control and analyzed using SPSS for windows version 16.0 (SPSS Chicago, IL.). Parametric (numeric) data were expressed as mean (SD) and compared using independent sample t-test. Non parametric data were expressed as frequency (number, percent) and compared using Mann- Whitney U test. Two tailed significant values were considered when p< 0.05.

## Results

A total of 50 children below 2 years of age fulfilled the protocol inclusion and exclusion criteria; were enrolled in this study. The initial demographic and clinical data of the two groups studied; 25 patients receiving milk formula with *Bifidobacterium Lactis* (*B. lactis* group) and 25 receiving milk formula with no *Bifidobacterium Lactis* (control group) were illustrated in Table 1.

**Table 1:** Study population baseline characteristics

	*B. lactis* group (n = 25)	Control group (n = 25)	*P* value
Age, months*	12.36 (6.07)	11.84 (6.56)	0.77
Gender**			
Male	15 (60)	16 (64)	0.77
Female	10 (40)	9 (36)	
Body weight, kg*	9.52 (2.67)	8.51 (2.10)	0.15
Duration of diarrhea before randomization, hours*	48.31 (9.44)	51.03 (8.16)	0.76
Children with fever**	7 (28)	6 (24)	0.63
Children with vomiting**	5 (20)	6 (24)	0.64

* mean(SD)

** number (%).

There were no significant differences (*P* > 0.05) between the two groups at randomization in terms of age at entry, birth weight, gender, breastfeeding before the study. The mean daily formula volume did not differ significantly between the control and *B. lactis* group (means [SD]: 589.4 [76.0] vs 578 [69.7] mL, respectively; *P* = 0.288). Furthermore, no significant differences were observed between groups in terms of other feeding characteristics (daily number of meals, regurgitation and vomiting episodes).

The mean duration (hours) of diarrhea before treatment was 48.31 ± 9.44 in (*B. lactis* group) and 51.03 ± 8.16 in (control group) (*P* = 0.76). Percentage of fever in *B. lactis* group was 28% and 24% in control group (*P* = 0.63). Percentage of vomiting in *B. lactis* group was 20% and 24% in control group (*P* = 0.64).

After 7 days of treatment the primary (frequency and duration of diarrhea, hospital stay) and secondary outcomes (frequency of vomiting and duration of fever) as illustrated in Table 2, indicated that the mean duration of diarrhea (days) after treatment was significantly shorter in children receiving *B. lactis* (*P* = 0.03).

**Table 2:** Primary and secondary outcome measures

	*B. lactis* group (n = 25)	Control group (n = 25)	*P* value
Duration of diarrhea after randomization, (days)*	3.12 ± 0.92	4.10 ± 0.94	0.02
Episodes of diarrhea /day*	3.96 (0.62)	4.46 (0.85)	0.04
Duration of hospital stay**			
< 2 days	18 (32)	11 (44)	
> 2 days	7 (28)	14 (56)	0.04
Episodes of vomiting / day*	3.21 (0.33)	3.55(0.06)	0.34
Duration of fever, days*	2.27 (0.85)	2.79 (0.64)	0.56

* mean (SD)

** number (%).

Frequency of diarrhea (number of motions per day) after treatment was significantly lower in children receiving *B. lactis* (*P* = 0.04). The duration of hospital stay was significantly less in children receiving *B. lactis*; 32% of children receiving *B. lactis* discharged form hospital before 2 days compared to 44% of control group (*P* = 0.04). There was no significant difference regarding reduction in episodes of fever and duration of vomiting in both groups. No adverse effects were noted in both groups.

## Discussion

This randomized double-blind controlled clinical trial demonstrated that for children under two years of age suffering from acute diarrhea, administration of 14.5 ×10^6^ CFU/100 ml daily of *Bifidobacterium Lactis* in a supplemented milk formula for 7 days results in less frequent episodes with shortening of the duration of diarrhea and less duration of hospital stay.

Probiotics have been most extensively studied in the treatment of diarrheal diseases, where their efficacy can be considered well established. Only a limited number of probiotic strains have been tested, and, as the effects of different probiotic microorganisms are not equivalent, results cannot be generalized [1].

There are many microorganisms that could potentially function as probiotics, of which *Lactobacillus* and *Bifidobacterium* species are the most commonly used. The genus *Bifidobacterium* includes various Gram positive non-motile anaerobic bacteria. They are endosymbiotic inhabitants of the gastrointestinal tract and vagina of mammals, including humans [23].

Strains of the genus *Bifidobacterium* are also often used as probiotic bacteria as they are known for their variety of resistance mechanisms to bile salts, which is important since the beneficial effects of probiotic bacteria must be generated in the presence of this biological fluid. It has even been proven that although bile tolerance is strain dependent, wild type-bile sensitive bifidobacteria strains can progressively adapt to the presence of bile salts by subculturing and gradually increasing concentration of bile [24].

In addition, non-pathogenic species belonging to the class of *Saccharomyces*, *Streptococcus* and *Lactococcus* are also used as probiotics [25]. Pathogens are microorganisms that may cause disease in their host. The ability to inhibit pathogens is one of the three main mechanisms of probiotics, barrier function enhancement and immune interactions being the other two. It is proposed that pathogen inhibition is facilitated through multiple mechanisms including: production of inhibitory substances (organic acids, H_2_O_2_, bacteriocins); nutrient competition; toxin removal/degradation; competition for sites of adherence (mucus, cell receptors); co-aggregation and virulence modulation; and induction of host immune responses by inducing immune-modulatory activity, including recruitment of CD4 + T-helper cells [26].

Of the studies on probiotics effect in children diarrhea; a Cochrane review [27] found that the use of *LGG* reduced the duration of diarrhea and mean stool frequency on day 2, while in a more recent systematic review [28] *LGG* had no effect on the total stool volume but significantly reduced the duration of diarrhea compared with placebo or no treatment. *LGG* was more effective when used at a daily dose of >10^10^ colony-forming units (CFU). Another Cochrane review [29] documented that the use of *S boulardii* reduced the risk of diarrhea lasting >4 days. The use of *S boulardii* significantly reduced both the duration of diarrhea and the risk of diarrhea on day 3. Similar results were found using *L reuteri* DSM 17938 [30].

Studies on *B lactis* are not frequent, one RCT [31] conducted on 224 Chinese children ages 6 to 36 months evaluated the effect of a lactose-free formula supplemented with 2 doses of a mixture of *B lactis* B12 and *S thermophilus* TH4 compared with unsupplemented formula. Regardless of the dose used, the duration of diarrhea was the same in both groups. In our study we used *B lactis* in a daily dose of 14.5 million CUF/100 ml and this dose was sufficient to induce reduction in frequency and duration of diarrhea.

In the present study, based on the average daily intake of formula, the mean daily ingested dose of *Bifidobacterium Lactis* was 14.6 ×10^8^ CFU/day, and according to a recent study, oral administration of lactobacilli at levels ranging from 10^8^ to 10^10^ CFU/day has led to transient colonization of the infant gastrointestinal tract [32]. Most of the stool pathogens in our series were probably viral. Stool analysis for rotavirus was positive in 66%, none of stool samples were positive for routine bacterial cultures. This finding is in accordance with many previous studies demonstrating that probiotic agents are able to treat intestinal infections, mainly of viral etiology [33, 34].

The mechanisms by which probiotic agents might exert their therapeutic effect against viral pathogens in particular are mostly unknown. An increased humoral response, including an increase in IgA-specific antibody-secreting cells against rotavirus, was described in children with acute rota viral diarrhea who received Lactobacillus GG [35]. Another study [36] showed that dietary treatment using *B. lactis* HN019 can reduce the severity of weanling diarrhea associated with rotavirus and *E. coli*, possibly via a mechanism of enhanced immune-mediated protection. That study suggested that probiotic treatment may be an effective dietary means of preventing or limiting diarrhea in human infants.

*Bifidobacterium lactis* was tested for prevention of diarrhea in a randomized control trial [37] of a commercial probiotic containing *Bifidobacterium lactis* and *S. thermophilus* involved 157 infants, 6 to 36 months of age. That study found a significant difference in the incidence of antibiotic-associated diarrhea in children receiving probiotic-supplemented formula (16%) compared with non-supplemented children. Our study was applied for children with acute viral diarrhea with exclusion of antibiotic associated causes.

As regarding safety, no adverse effects were noted in our study. Bifidobacteria are generally regarded as nonpathogenic, because they occur naturally in the intestine. In many clinical trials these agents seem to be safe for the general pediatric populations and in particular in infancy [38].

Depending on the presence of great variety of species and strain characteristics of probiotics, there is always a need for more controlled clinical studies investigating new types of probiotics.

We concluded that *Bifidobacterium lactis* probiotics in supplemented milk formula decreased significantly frequency, duration of diarrhea, and hospital stay than usual treatment alone in children with acute diarrhea. Additional researches on other uncommon local probiotic species should be encouraged.
